# Concentrating childhood cancer treatment in the Netherlands

**DOI:** 10.1007/s00608-015-0282-3

**Published:** 2015-08-26

**Authors:** Hans van Goudoever

**Affiliations:** Emma Children’s Hospital AMC Amsterdam, VU University Medical Center, Boelelaan 1117, PO Box 7057, 1007 MB Amsterdam, The Netherlands

**Keywords:** Paediatrics, Centralized care, Concentration of care, Paediatric oncology, Netherlands, Pädiatrie, Zentralisierte Gesundheitsversorgung, Konzentration der Gesundheitsversorgung, Pädiatrische Onkologie, Niederlande

## Abstract

Paediatric tertiary care is highly centralized in the Netherlands. The country is small (16 million inhabitants, overall unemployment rates were approximately 7 % in 2014, while young adult (< 25 years) unemployment rates were 12 %) with the majority of the population living in Amsterdam and Rotterdam and their neighbouring cities/villages. There are 90 hospitals taking care for children in the Netherlands. Specific types of highly specialized care, such as transplantation, are provided in a maximum of three centres (kidney transplants in 3, bone marrow tranplants in 2, liver transplants in only one center), while neonatal intensive care is offered in 10 hospitals. Recently, patients with solid tumours in the thorax and abdomen were concentrated in a single centre with five university centres who provide care during the less intensive part of the treatment (in shared care). Similar changes are planned for congenital surgery, aiming for two such centres in the Netherlands. The general view of the Dutch Paediatric Association underscores the need for centralisation, while high-level care should be guaranteed at those hospitals where no specialized centre is present.

## Zusammenfassung

Die tertiäre Krankenversorgung für Kinder ist stark zentralisiert in den Niederlanden. Das Land ist klein (16 Mio. Einwohner, die Arbeitslosenquote 2014 lag schätzungsweise bei 7 %, unter jungen Erwachsenen (< 25 Jahre) bei 12 %) und die Hälfte der Bevölkerung lebt in Amsterdam und Rotterdam sowie den benachbarten Städten bzw. Dörfern. Fast 90 Krankenhäuser leisten die stationäre Gesundheitsversorgung für Kinder. Bestimmte Arten hochspezialisierter Behandlungen wie Transplantationen sind nur in höchstens drei Klinikzentren verfügbar (Nierentransplantationen in 3, Knochenmarktransplationen in 2, Lebertransplantationen nur in einem Zentrum), während z. B. die Neugeborenenintensivmedizin in zehn Krankenhäusern angeboten wird. Neuerdings werden Patienten mit soliden Tumoren in Thorax und Abdomen in einem einzigen Zentrum behandelt gemeinsam mit 5 Universitätskliniken, die sich die Versorgung während der weniger intensiven Behandlungsperiode teilen. Für die Chirurgie angeborener Fehlbildungen sind ähnliche Veränderungen geplant. Es werden zwei solcher Zentren für die Niederlande anvisiert. Die niederländische Gesellschaft für Kinder- und Jugendmedizin unterstreicht die Notwendigkeit der Zentralisierung, während die generelle Versorgung auf höchstem fachlichem Niveau in den Krankenhäusern garantiert sein sollte, welche keine spezialisierten Zentren aufweisen.

## Introduction

The Netherlands is one of the smaller European countries with approximately 16.5 million inhabitants. Paediatric tertiary care is centralized in eight university medical centres, while another 80 hospitals taking care for children are spread across the country. The time required to travel to the closest hospital is within 60 minutes for more than 99 % of the population. Health care is provided free of charge for children as they are covered by their parents’ health insurance. Furthermore, schools are also free. The number of private schools is very small. University fees are € 1,900 annually. Overall unemployment rates were approximately 7 % in 2014, while young adult (< 25 years) unemployment rates were 12 %.

Paediatric care, and especially tertiary care, is highly centralized (Tab. 1). This article focusses on the process of concentrating childhood cancer in the Netherlands.

**Table 1:** 

Liver, small intestine, lung transplantation	1 centre
Bone marrow transplantation	2 centres
Kidney transplantation	3 centres
Cardiac surgery	4 centres
Oncology	7 centres
Paediatric intensive care	8 centres
Neonatal intensive care	10 centres

## Childhood cancer

**Figure 1:**
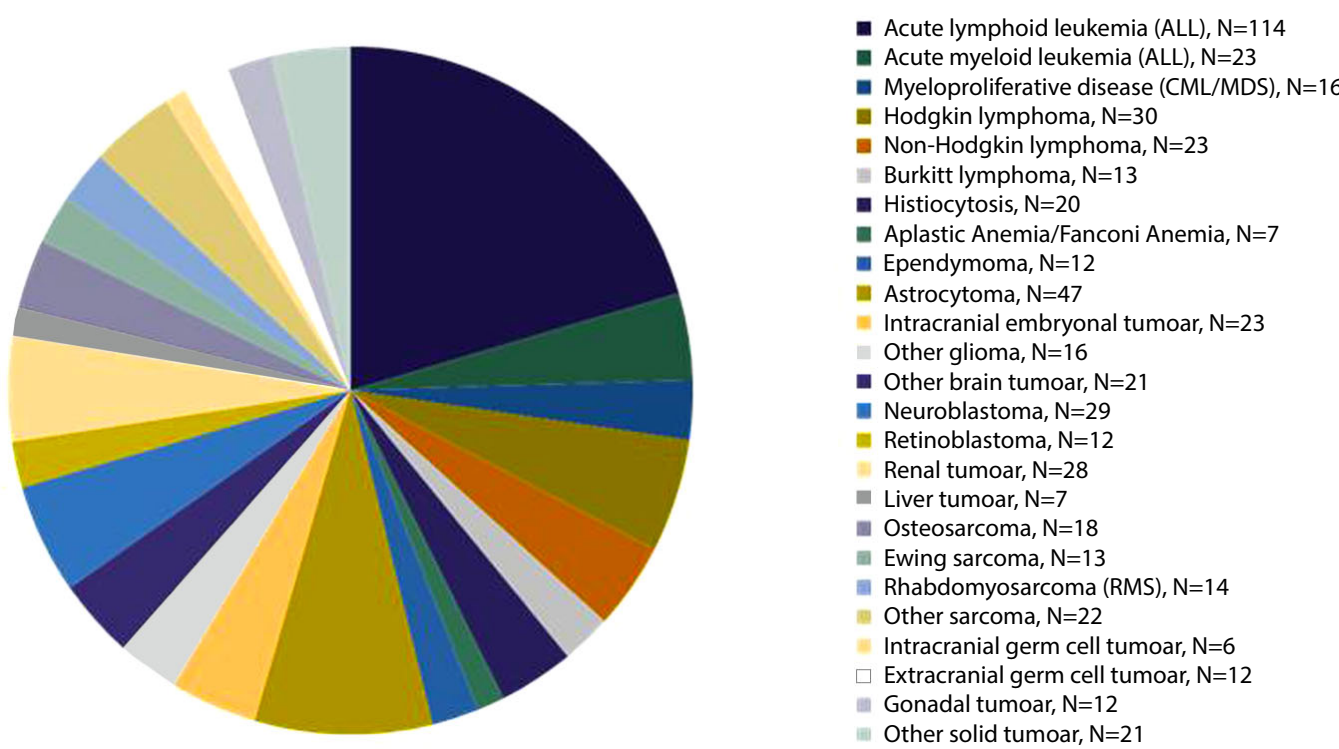

**Figure 2:**
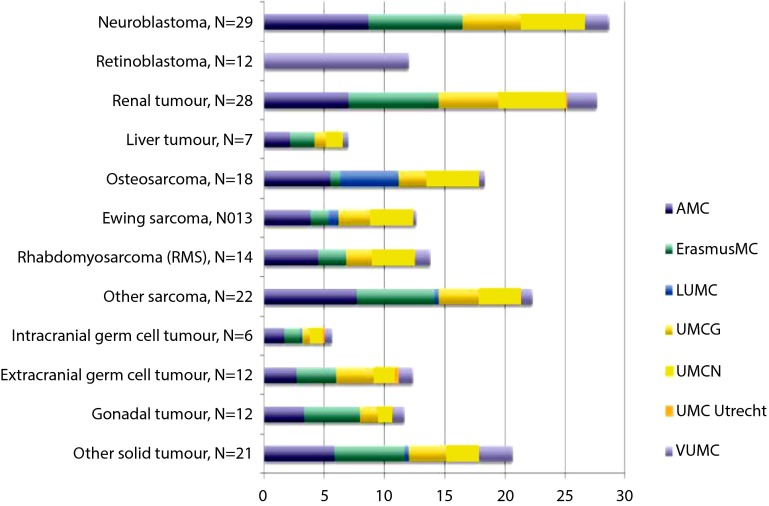

Approximately 550 new cases of cancer are diagnosed each year in the Netherlands. Many types of cancer occur less than 25 times per year. While cancer is a frequent cause of death, the overall survival rate is 75%. The distribution of the different types of childhood malignancies is shown in Fig. 1. For several types of childhood cancer, the number of patients seen at the present centres is low. An example is shown in Fig. 2, where the prevalence of the paediatric patients with solid tumours are presented for the seven university medical centres in the Netherlands. The largest, the Amsterdam Medical Center, hosts approximately 30 children each year, while the smallest centres treat lees than 10 patients each year.

For several adult cancer types, there is evidence that treatment in high-volume hospitals or by high case-volume providers leads to a better outcome compared with low-volume hospitals or low case-volume providers [1, 2]. As an explanation for this positive correlation, the “practice makes perfect” effect has been used; a greater supply of patients will give physicians and their teams more experience and will consequently lead to improvement of care. Another explanation of this volume effect is the phenomenon of selective referral, which means a physician or hospital with a reputation of excellent care will attract more patients. In a recent review, Knops et al. provides support for the statement that higher-volume hospitals, higher case-volume providers, and specialised hospitals are related to better outcomes in paediatric oncology [3]. However, this is challenged in a recent retrospective study from Switzerland [4]. Joseph et al. showed excellent survival and morbidity rates in abdominal and thoracic paediatric solid-tumour surgery in low volume centers.

Despite the contrasting data, paediatric oncologists, together with a parent paediatric oncology association in the Netherlands, decided that all paediatric cancer patients should be diagnosed in a single centre. Following a bidding process that caused a lot of collateral damage, Utrecht was chosen to be that centre. It was decided that the initial treatment should be started there as well. Subsequent therapy could be provided in that centre or in a limited number of other hospitals.

So far, only abdominal and thoracic solid-tumour treatments are centralized, although patients with retinoblastoma are treated in a single centre as well, although not in Utrecht but in Amsterdam (VU University Medical Center international (VUmc), Amsterdam, Netherlands).

## Advantages of concentrating childhood cancer care

Despite the discussion on the presupposition that higher volume would result in better outcome, most experts underline this statement whenever surgical care is involved. Other advantages include the possibility of specific treatments for small subsets of patients, where recognition of rare deteriorations following therapy might be noted earlier. Costs and efficiency are mentioned as well. As such, a centre hosts all national experts and may be attractive to international experts as well. Large numbers of patients will be cared for and large international multicentre research studies are likely to be carried out at this centre. Training and education can be developed easily, as many types of care are centralized in such a centre. Finally, this cancer hospital may be considered an international centre of reference, although that kind of recognition will depend on the results obtained.

## Disadvantages of a single national paediatric cancer centre

As the expectations are high, the demands are high as well. The costs of the new paediatric cancer centre are estimated at € 190,000,000, while most Dutch oncology wards at the different university medical centres are already state of the art. That implies a considerable amount of wasted investments. Disturbances in relations between professionals and parents (loss of trust) or between healthcare professionals themselves implies that patients and professionals will have to go abroad to seek treatment or employment, respectively. Interaction with the professionals who take care of adult patients should be sought actively, whereas the interaction occurs more naturally in the present setting because of historical ties. Training young paediatricians will be difficult as well. With a lack of exposure, many paediatricians will be trained without being in contact with children with cancer. This can be solved, for example, by 3-month internships, but this requires the young doctors to move for that period.

## Centralized research

Obviously there are many advantages in treating all paediatric patients at one hospital. All patients can easily be asked for consent. Efficiency and fundraising possibilities will also be improved. In spite of seeking sponsors in the region, a national programme can be developed. Disadvantages include a lack of national competition for obtaining research funding and dominance by certain groups in the field of pediatric oncology research as no competative groups are exisiting.

## The Dutch model of centralized paediatric oncology

We started earlier this year by bringing all children with solid abdominal and thoracic tumours to the Prinses Maxima Centre in Utrecht, which is a department of the children’s hospital for the next 3–4 years. Collaboration exists with the existing paediatric oncology centres that are currently treating more than 85 % of all paediatric oncology patients. The collaboration means that patients are treated in shared care. The initial chemotherapy and surgery occurs in Utrecht, but after this, the children are treated alternately at both Utrecht and a university hospital in the region of the family. It is also possible to have shared care with a non-university regional hospital.

Plans are being developed to organize the care in a similar fashion for some but not all other types of childhood cancer. Within this model, the shared care is supervised by the centre in Utrecht. This process is cumbersome, as many stakeholders are involved and improvement in care for a specific group should never lead to deterioration of the quality of care for another group. Also the transition is a difficult period, during which it is difficult to keep the quality of care at the same level. New teams are being formed, which inevitably requires some time to adapt.

## Aims

The aims are high. The board of the Prinses Maxima Centre strives for a cure rate of > 90 % by 2025, with less than half of the patients suffering from late effects of treatment. Those goals are ambitious, but certainly worth aiming for. The future will tell whether this can (and should) be reached through this kind of centralization.
